# Historical Redlining, Contemporary Gentrification, and Severe Maternal Morbidity in California, 2005-2018

**DOI:** 10.1001/jamanetworkopen.2024.29428

**Published:** 2024-09-23

**Authors:** Xing Gao, Rachel Morello-Frosch, Amani M. Nuru-Jeter, Jonathan M. Snowden, Suzan L. Carmichael, Mahasin S. Mujahid

**Affiliations:** 1 Department of Obstetrics, Gynecology & Reproductive Sciences, University of California, San Francisco; 2 Department of Environmental Science, Policy and Management, University of California, Berkeley; 3 Division of Environmental Health Sciences, School of Public Health, University of California, Berkeley; 4 Division of Community Health Sciences, School of Public Health, University of California, Berkeley; 5 Division of Epidemiology, School of Public Health, University of California, Berkeley; 6 School of Public Health, Oregon Health & Science University-Portland State University; 7 Department of Obstetrics & Gynecology, Division of Maternal-Fetal Medicine, Stanford University, Stanford, California; 8 Department of Pediatrics, Division of Neonatal & Developmental Medicine, Stanford University, Stanford, California

## Abstract

**Importance:**

Historically redlined neighborhoods may experience disinvestment, influencing their likelihood of gentrification, a process of neighborhood (re-)development that unequally distributes harms and benefits by race and class. Understanding the combined outcomes of redlining and gentrification informs how the mutually constitutive systems of structural racism and racial capitalism affect pregnancy outcomes.

**Objective:**

To examine if historical redlining and contemporary gentrification is associated with increased severe maternal morbidity (SMM) odds.

**Design, Setting, and Participants:**

This cross-sectional study used data from a statewide population-based sample of all live hospital births at 20 weeks’ gestation or more between 2005 and 2018 in California. Analysis was conducted from March 2023 to January 2024.

**Exposure:**

Redlining (as characterized by the federal Home Owners’ Loan Corporation mortgage security maps) and displacement (using present-day sociodemographic and housing market information).

**Main Outcomes and Measures:**

Mixed-effects logistic regression models were used to assess the association of census tract–level exposure to historical redlining and contemporary gentrification with increased SMM odds, adjusting for sociodemographic and pregnancy related factors. Outcome classification was based on the Centers for Disease Control and Prevention SMM index, which defines SMM as having any of the 21 procedures and diagnoses based on the *International Classification of Diseases, Ninth Revision* and *International Statistical Classification of Diseases and Related Health Problems, Tenth Revision* codes.

**Results:**

The study sample included 1 554 837 births (median [SD] maternal age, 29.0 [6.4] years; 3464 American Indian or Alaskan Native [0.2%], 224 774 Asian [14.5%], 132 240 Black [8.5%], 880 104 Hispanic [56.6%], 312 490 White [20.1%]), with 22 993 cases of SMM (1.4%). Residents in historically redlined neighborhoods that were undergoing gentrification or displacement were more likely to be Black, Hispanic, and American Indian or Alaskan Native. Independent of individual-level characteristics, SMM odds were greater for individuals living in redlined neighborhoods that experienced displacement (OR, 1.21; 95% CI, 1.14-1.28) and in redlined neighborhoods undergoing gentrification (OR, 1.21; 95% CI, 1.13-1.29) compared with those in continuously advantaged neighborhoods.

**Conclusions and Relevance:**

Findings from this cross-sectional study demonstrate that the legacies of redlining, intertwined with current dynamics of displacement and gentrification, affect SMM. Place-based sociopolitical mechanisms that inequitably distribute resources may be important intervention points to address structural drivers of adverse pregnancy outcomes and their racial inequities.

## Key Points

**Question:**

Is living in a historically redlined neighborhood currently experiencing gentrification associated with the odds of experiencing severe maternal morbidity (SMM)?

**Findings:**

In this cross-sectional study of a population-based sample of all live hospital births in California, living in a historically redlined neighborhood that currently experiences either displacement or gentrification was associated with greater odds of SMM for birthing people compared with residency in continuously advantaged neighborhoods.

**Meaning:**

These findings reinforce the importance of addressing past and present mechanisms that shape neighborhood social and material conditions to advance the well-being of birthing people in California.

## Teaser

This cross-sectional study of live hospital births in California examines the association of historical redlining, displacement, and gentrification with increased odds of severe maternal morbidity.

## Introduction

The inequitable distribution of health-promoting resources along racial and class lines produces persistent inequities in pregnancy-related mortality and morbidity outcomes.^1,2,3^ This geography of inequality has been shaped by discriminatory practices across multiple sociopolitical systems and economic institutions.^4,5,6^ Structural racism, defined as the totality of racist practices and policies embedded in societal institutions, and racial capitalism, or the accumulation of profit through the racialized exploitation and devaluation of marginalized people across institutions, are mutually constitutive systems that rely on and enforce socially produced differences, specifically the racial hierarchy.^5,7,8,9,10^ Discriminatory and profit-driven place-based mechanisms often concentrate advantages in areas with predominantly White and upper-class residents while simultaneously extracting economic value from racially marginalized, working-class populations and their neighborhoods.^4,9,10^

Historical redlining exemplifies the lasting influence of structural racism in policymaking. The federal Home Owners’ Loan Corporation (HOLC), a New Deal initiative to provide relief to mortgage holders at risk of foreclosure, created maps of lending risk in neighborhoods.^11^ Areas with higher proportions of people of color, immigrants, and working-class residents were assigned red high-risk grades (ie, redlining).^11,12,13,14^ Mortgage redlining was later adopted by other governmental agencies, acting in concert with discriminatory housing practices carried out by public and private agents.^12,13^ This set the stage for the deaccumulation of protective resources and hyperaccumulation of risk in predominantly non-White neighborhoods.^15^ Today, redlined neighborhoods have more environmental hazards, fewer amenities, and reduced accumulation of generational wealth, increasing the risk of adverse pregnancy-related outcomes.^4,16,17,18,19,20,21^

Neighborhoods with a history of housing discrimination may be vulnerable to gentrification.^22,23,24^ Through the lens of racial capitalism, neighborhood gentrification is a racialized and profit-driven process in which an influx of public and private investment targets disinvested neighborhoods, often with higher concentrations of residents of color, for redevelopment.^25^ Gentrification can result in better infrastructure and amenities, which support health-promoting resources during pregnancy. However, gentrification can also lead to the rising cost of living, disrupting community support and creating psychosocial stressors, increasing the risk of adverse pregnancy outcomes.^26,27,28^ The distribution of harms and benefits associated with gentrification is often unequal along axes of social stratification.^29,30,31,32,33,34,35^

Severe maternal morbidity (SMM), defined as unexpected and life-threatening complications that occur before, during, or after delivery, has been adopted to characterize birthing people’s well-being.^36,37,38^ The 3-fold increase in SMM rates over the last decade is accompanied by widening racial and ethnic inequities.^36,39,40,41,42,43,44^ Individual-level factors have not sufficiently explained the increasing occurrence of SMM and its inequities, demonstrating the need to examine contextual factors.^45,46,47,48,49,50^ Existing research has documented that residing in a previously redlined or a currently gentrifying neighborhood may be associated with increased risks of adverse pregnancy-related and infant outcomes, but the combined influence of redlining and gentrification on pregnancy-related mortality and morbidity remains unknown.^18,20,35,51,52^ Connecting racialized historical disinvestment—exemplified by HOLC redlining—to contemporary extraction of value from marginalized neighborhoods through gentrification-related development creates an opportunity to make tangible the structural forces that operate across time and space.^25,53^ This connection illuminates the repeated and sequential nature of these inequitable policies and programs, informing future efforts to center equity in place-based development.

This investigation aims to assess the association place-based manifestations of structural racism and racial capitalism, or the combined influence of historical redlining and contemporary gentrification with SMM and related racial inequities. We hypothesized that living in a neighborhood exposed to sequential redlining and gentrification would be associated with greater odds of SMM, compared with living in a neighborhood that did not experience redlining and is currently economically advantaged. Furthermore, we hypothesized that this association would be more pronounced among racially and ethnically marginalized groups.

## Methods

### Study Population

The population for this cross-sectional study consisted of all live singleton births in California hospitals between 2005 and 2018, sourced from the Department of Health Care Access and Information. Data-linked birth certificates with parent and infant hospital discharge records, which included the *International Classification of Diseases, Ninth Revision, Clinical Modification (ICD-9-CM)* and *International Statistical Classification of Diseases, Tenth Revision, Clinical Modification (ICD-10-CM)* codes. Birth cohort files included the birthing person’s geocoded residential address at birth, which was assigned census tract identifiers to link to exposure variables.

From 6 753 639 total births, we excluded births that could not be linked to a census tract, had missing covariate information, were plural birth type, or had missing or implausible gestational age. The HOLC created maps for cities with more than 40 000 residents during 1930 to 1940, which included 8 California cities: Fresno, Los Angeles, Oakland, Sacramento, San Diego, San Francisco, San Jose, and Stockton. Births outside of historical redlining map coverage were excluded due to missing exposure information. We also excluded births that did not have gentrification information. The final analytic sample included 1 554 837 births (eFigure in Supplement 1). The California Committee for the Protection of Human Subjects and the institutional review boards of Stanford University and the University of California, Berkeley approved the study protocol; informed consent was not required because data were deidentified. This study follows the Strengthening the Reporting of Observational Studies in Epidemiology (STROBE) reporting guideline for observational studies.

### Outcome

SMM from delivery hospitalization through 42 days postpartum was defined using an index developed by the Centers for Disease Control and Prevention, validated for use with administrative data.^40,54,55^ The SMM index is based on 21 potentially fatal conditions and life-saving procedures related to pregnancy, labor, or delivery, identified using *ICD-9-CM* and *ICD-10*-*CM* diagnosis and procedure codes (eTable 1 in Supplement 1). Individuals with at least 1 of these 21 indicators were classified as cases. For the sensitivity analysis, we assessed nontransfusion SMM, which excludes cases that only had a blood transfusion indicator. Due to the lack of information on the volume of transfusion in administrative data, including cases with blood transfusion as the sole indicator may include cases with only low-volume transfusion, resulting in overestimation of SMM cases.^44,54,56^

### Exposure

#### Redlining

Historical redlining was assessed using digitized HOLC maps.^11^ Maps of 8 California cities were digitized into shapefiles. HOLC grades included: “A: Best,” “B: Still Desirable,” “C: Declining,” and “D: Hazardous.”^11^ We overlaid HOLC grades with census tract boundaries from the 2000 census for births between 2005 and 2009, as well as with census tract boundaries from the 2010 census tracts for births between 2010 and 2018.^17^ We used an area-weighted method for best alignment with the census tract boundary.^57,58^ Based on the tract percentage of land within or overlapping with each type of HOLC grade, we weighted the grade by the percentage of land area. This continuous score was rounded to 4 categories corresponding to HOLC grades. Due to sample size and the assessment of a rare outcome, we created a dichotomous measure of redlining (tracts graded C and D), and nonredlined, advantaged neighborhoods (tracts graded A and B).

#### Gentrification

Gentrification was assessed using the Displacement and Gentrification Typology (DGT), which tracks neighborhood investment and sociodemographic changes.^28,59,60^ DGT captures how affordable the local housing market is for low-, middle-, and high-income families as a key feature of gentrification, which may be especially salient for SMM outcomes.^52^ This measure also has the advantage of characterizing the displacement of low-income residents, a stage of neighborhood change in economically disadvantaged neighborhoods that may precede, accompany, or follow gentrification and its associated increase in housing cost.^61^

Neighborhoods were classified into 9 categories, collapsed into 3 broad stages: *displacement*, which characterizes economically disadvantaged, low-income neighborhoods that may be susceptible to or experiencing the loss of low-income households; *gentrification*, defined as low- or mixed moderate– and higher-income neighborhoods that experience a rise in housing costs; and *exclusive*, or neighborhoods that are socioeconomically advantaged and inaccessible to low- and middle-income families (eTable 2 in Supplement 1).

We measured gentrification across two 10-year periods normalized to 2010 census tract boundaries.^62^ We linked births to their respective period with a 5-year lag between the beginning of the period and birth years to increase the likelihood that the measured neighborhood changes have been occurring when the birth occurred (eTable 3 in Supplement 1).^62,63^ The first period from 2000 to 2010 used the 2000 Decennial Census and the 2008-2012 American Community Survey 5-Year Estimate. The second period from 2007 to 2018 used the 2005-2009 and 2015-2019 American Community Survey 5-Year Estimate. For specific criteria, the tract-level characteristics were compared with the corresponding regional characteristics at the core-based metropolitan and micropolitan statistical areas level (ie, tract rent increase compared to regional rent increase).

#### Composite Measure

To assess the combined influence of historical redlining and present-day gentrification on SMM, we created a measure that integrated the HOLC grades with the DGT-measured gentrification classification. Individuals who resided in exclusive neighborhoods (not undergoing displacement or gentrification) that did not experience redlining, or areas with continuous advantage, were used as the referent group. The composite measure included the following categories: (1) not redlined and exclusive (referent), (2) not redlined and undergoing displacement, (3) not redlined and undergoing gentrification, (4) redlined and exclusive, (5) redlined and undergoing displacement, and (6) redlined and undergoing gentrification.

### Covariates

Covariates included the birthing person’s age (years), education (less than high school, high school, some college, college or postgraduate degree), source of payment at delivery (private insurance, Medicaid, uninsured or other), and parity (any or no prior live births).

Race and ethnicity categories, assessed using self-reported information on birth certificates, included non-Hispanic American Indian or Alaska Native, Asian or Pacific Islander, Black, Hispanic, and White. We position race and ethnicity as a proxy that characterizes past and present structural racism that racially marginalized people have experienced.^64,65^

### Statistical Analysis

We examined the distribution of the participants’ characteristics by SMM status and exposure status, as well as the distribution of exposure to composite redlining and gentrification by race and ethnicity. We used mixed-effects logistic regression, with a random intercept to account for participants clustered within neighborhoods, to assess associations between the composite redlining and gentrification exposure and SMM.^66^ We sequentially adjusted for covariates—model 1 adjusted for age, model 2 further adjusted for educational attainment and insurance type, and model 3 additionally adjusted for parity—to investigate how redlining and gentrification were associated with SMM independent of individual socioeconomic and pregnancy factors. We assessed effect measure modification by race and ethnicity using an interaction term between the combined redlining and gentrification measure with race and ethnicity.^67,68^ Sensitivity analysis assessed nontransfusion SMM as the outcome. Statistical significance testing used 95% CI, corresponding to a 2-sided α level of .05. Analyses were conducted in Stata IC version 15.1 (StataCorp LLC).

## Results

In a sample of 1 554 837 live singleton births, 21 788 were classified as SMM cases (1.4%). There were 2344 tracts, with a mean (SD) 673 (397.4) births per tract (range, 1-2244 births). The mean (SD) age was 29.0 (6.4) years. Individuals in redlined and displacement tracts, as well as those in redlined and gentrifying tracts, were more likely to have experienced SMM (redline and displacement, 9376 of 21 788 [43.0%] vs 620 593 of 1 533 049 [40.5%]; redlined and gentrifying, 3086 of 21 788 [14.2%] vs 204 893 of 1 533 049 [13.4%]) (Table 1). SMM prevalence was the lowest in nonredlined and exclusive tracts (2499 of 207 787 [1.2%]) and the highest in redlined areas undergoing displacement (151 of 10 827 [1.5%]) (Table 2). In American Indian and Alaskan Native, Hispanic, and White groups, SMM prevalence was the highest for those in redlined tracts undergoing gentrification at 2.9% (under 12 cases), 1.5% (2101 cases), and 1.3% (238 cases), respectively. Among Black individuals, the highest prevalence of SMM was among those in redlined tracts undergoing displacement, or 2.2% (1587 cases) (Figure). In nonredlined and exclusive tracts, or areas with continous advantage, Black birthing people had 2.1% SMM prevalence (238 cases) compared with 1.0% SMM prevalence (983 cases) for White birthing people.

**Table 1.** Participant Characteristics by Severe Maternal Morbidity Status, California, 2005-2018

Characteristic	Overall, No. (%) (N = 1 554 837)^a^	Birthing persons, No. (%)	
		No SMM (n = 1 533 049)	SMM (n = 21 788)
Redlining + Displacement and Gentrification Typology^b^			
Nonredlined + displacement	57 064 (3.7)	56 307 (3.7)	757 (3.5)
Nonredlined + gentrification	10 827 (0.7)	10 676 (0.7)	151 (0.7)
Nonredlined + exclusive	207 787 (13.4)	205 288 (13.4)	2499 (11.5)
Redlined + displacement	629 969 (40.5)	620 593 (40.5)	9376 (43.0)
Redlined + gentrification	207 979 (13.4)	204 893 (13.4)	3086 (14.2)
Redlined + exclusive	441 211 (28.4)	435 292 (28.4)	5919 (27.2)
Age, y			
<20	120 285 (7.7)	118 437 (7.7)	1848 (8.5)
20-34	1 098 637 (70.7)	1 084 399 (70.7)	14 238 (65.3)
≥35	335 915 (21.6)	330 213 (21.5)	5702 (26.2)
Race or ethnicity			
American Indian or Alaskan Native	3464 (0.2)	3396 (0.2)	68 (0.3)
Asian or Pacific Islander	224 774 (14.5)	221 669 (14.5)	3105 (14.3)
Black	132 240 (8.5)	129 441 (8.4)	2799 (12.8)
Hispanic	880 104 (56.6)	867 833 (56.6)	12 271 (56.3)
White	312 490 (20.1)	308 978 (20.2)	3512 (16.1)
Other^c^	1765 (0.1)	1732 (0.1)	33 (0.2)
Education			
Less than high school	424 556 (27.3)	418 175 (27.3)	6381 (29.3)
High school	373 600 (24.0)	368 170 (24.0)	5430 (24.9)
Some college	320 973 (20.6)	316 547 (20.6)	4426 (20.3)
College or graduate school	435 708 (28.0)	430 157 (28.1)	5551 (25.5)
Payment type at delivery			
Private	651 952 (41.9)	643 462 (42.0)	8490 (39.0)
Medi-Cal	842 771 (54.2)	830 193 (54.2)	12 578 (57.7)
Uninsured or other	60 114 (3.9)	59 394 (3.9)	720 (3.3)
Primiparous	638 711 (41.1)	628 675 (41.0)	10 036 (46.1)

Abbreviation: SMM, severe maternal morbidity.

^a^ Births outside of Home Owners’ Loan Corporation (HOLC) map coverage were not included in this study sample.

^b^ Historically redlined neighborhoods received a HOLC grade of A or B, nonredlined neighborhoods a C or D. The Displacement and Gentrification Typology was used to define displacement (low income or susceptible to displacement, ongoing displacement of low-income households), gentrification (at risk of gentrification, early ongoing gentrification, advanced gentrification), and exclusive (stable moderate or mixed income, at risk of becoming exclusive, becoming exclusive, stable or advance exclusive).

^c^ Other race included individuals reporting ≥2 racial groups or selecting “other” race or ethnicity.

**Table 2.** Gentrification and Redlining Status by Demographic Characteristics, California, 2005-2018

Characteristic	Overall, No. (%) (N = 1 554 837)^a^	Birthing persons, No. (%)^b^					
		Nonredlined + displacement (n = 57 064)^c^	Nonredlined + gentrification (n = 10 827)	Nonredlined + exclusive (n = 207 787)	Redlined + displacement (n = 629 969)	Redlined + gentrification (n = 207 979)	Redlined + exclusive (n = 441 211)
Age, y							
<20	120 285 (7.7)	5497 (9.6)	579 (5.3)	5341 (2.6)	68 907 (10.9)	19 318 (9.3)	20 643 (4.7)
20-34	1 098 637 (70.7)	42 352 (74.2)	7746 (71.5)	128 120 (61.7)	467 503 (74.2)	151 947 (73.1)	300 969 (68.2)
≥35	335 915 (21.6)	9215 (16.1)	2502 (23.1)	74 326 (35.8)	93 559 (14.9)	36 714 (17.7)	119 599 (27.1)
Race or ethnicity							
American Indian or Alaskan Native	3464 (0.2)	118 (0.2)	NR	439 (0.2)	1475 (0.2)	347 (0.2)	1074 (0.2)
Asian or Pacific Islander	224 774 (14.5)	3251 (5.7)	2698 (24.9)	43 797 (21.1)	57 017 (9.1)	28 648 (13.8)	89 363 (20.3)
Black	132 240 (8.5)	7293 (12.8)	687 (6.3)	11 604 (5.6)	72 596 (11.5)	15 760 (7.6)	24 300 (5.5)
Hispanic	880 104 (56.6)	40 648 (71.2)	5395 (49.8)	53 199 (25.6)	450 787 (71.6)	144 828 (69.6)	185 247 (42.0)
White	312 490 (20.1)	5656 (9.9)	2025 (18.7)	98 459 (47.4)	47 388 (7.5)	18 209 (8.8)	140 753 (31.9)
Other^d^	1765 (0.1)	98 (0.2)	NR	289 (0.1)	706 (0.1)	187 (0.1)	474 (0.1)
Education							
Less than high school	424 556 (27.3)	17 769 (31.1)	2306 (21.3)	15 210 (7.3)	250 363 (39.7)	75 173 (36.1)	63 735 (14.4)
High school	373 600 (24.0)	17 768 (31.1)	2460 (22.7)	25 959 (12.5)	184 619 (29.3)	57 748 (27.8)	85 046 (19.3)
Some college	320 973 (20.6)	13 334 (23.4)	2352 (21.7)	38 907 (18.7)	126 194 (20.0)	42 328 (20.4)	97 858 (22.2)
College or graduate school	435 708 (28.0)	8193 (14.4)	3709 (34.3)	127 711 (61.5)	68 793 (10.9)	32 730 (15.7)	194 572 (44.1)
Payment type at delivery							
Private	651 952 (41.9)	17 572 (30.8)	5148 (47.5)	155 795 (75.0)	153 066 (24.3)	57 125 (27.5)	263 246 (59.7)
Medi-Cal	842 771 (54.2)	38 020 (66.6)	5177 (47.8)	43 563 (21.0)	457 662 (72.6)	143 555 (69.0)	154 794 (35.1)
Uninsured or other	60 114 (3.9)	1472 (2.6)	502 (4.6)	8429 (4.1)	19 241 (3.1)	7299 (3.5)	23 171 (5.3)
Primiparous	638 711 (41.1)	21 479 (37.6)	4963 (45.8)	98 736 (47.5)	226 423 (35.9)	80 166 (38.5)	206 944 (46.9)
Severe maternal morbidity prevalence	21 788 (1.4)	757 (1.3)	151 (1.5)	2499 (1.2)	9376 (1.5)	3086 (1.5)	5919 (1.3)

^a^ Births outside of Home Owners’ Loan Corporation (HOLC) map coverage were not included in this study sample.

^b^ Historically redlined neighborhoods received a HOLC grade of A or B, nonredlined neighborhoods a C or D. The Displacement and Gentrification Typology was used to define displacement (low income or susceptible to displacement, ongoing displacement of low-income households), gentrification (at risk of gentrification, early ongoing gentrification, advanced gentrification), and exclusive (stable moderate or mixed income, at risk of becoming exclusive, becoming exclusive, stable or advance exclusive).

^c^ Not reported (NR) values indicate sample sizes with 12 or fewer observations, per data use agreement with California.

^d^ Other race included individuals reporting ≥2 racial groups or selecting “other” race or ethnicity.

**Figure.**
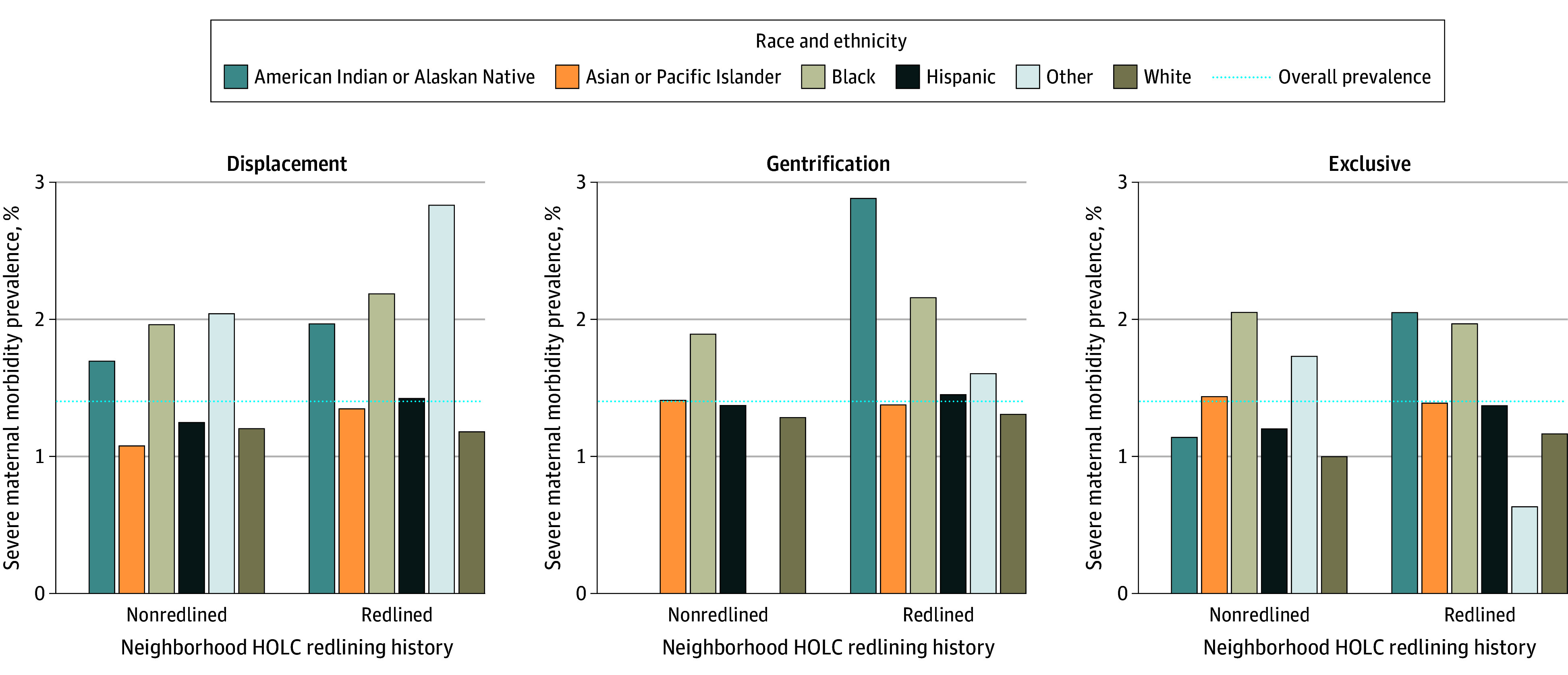
Race- and Ethnicity-Specific Severe Maternal Morbidity Prevalence by Gentrification and Redlining Status, 2005-2018 HOLC indicates Home Owners’ Loan Corporation. Historically redlined neighborhoods received a HOLC grade of A or B, nonredlined neighborhoods a C or D. The Displacement and Gentrification Typology was used to define displacement (having low income or susceptible to displacement, ongoing displacement of low-income households), gentrification (at risk of gentrification, early ongoing gentrification, advanced gentrification), and exclusivity (stable moderate or mixed income, at risk of becoming exclusive, becoming exclusive, stable or advanced exclusive). Prevalence for American Indian and Alaskan Native and other race individuals in nonredlined gentrification tracts not available due to insufficient sample size. Births outside of HOLC map coverage were not included in this study sample.

White birthing people were more likely to live in redlined and nonredlined exclusive neighborhoods (Table 2). Black and Hispanic birthing people were more frequently living in neighborhoods undergoing displacement, regardless of these neighborhoods’ redlining history. Additionally, a higher proportion of Hispanic individuals were living in redlined tracts experiencing gentrification. Those with public insurance and who had lower education attainment more frequently lived in redlined tracts undergoing displacement and gentrification. The majority of the neighborhoods currently undergoing displacement or gentrification were previously graded C or D (eTable 4 in Supplement 1). A very small study sample resided in non-redlining neighborhoods experiencing displacement (3.7%) and gentrification (0.7%).

Results from mixed effects models assessing associations between the combined exposure of gentrification and redlining with SMM (adjusted for sociodemographic factors and parity) showed that the effect size was the greatest between exposure to both redlining and gentrification (OR, 1.21; 95% CI, 1.13-1.29), followed by living in a historically redlined tract that continues to be economically disadvantaged and experiences displacement (OR, 1.21; 95% CI, 1.14-1.28) (Table 3). Living in tracts that experienced redlining but are now exclusive was also associated with increased odds of SMM (OR, 1.12; 95% CI, 1.06-1.19).

**Table 3.** Adjusted Odds Ratios of Severe Maternal Morbidity by Redlining and Gentrification in California, 2005-2018

Neighborhood classification^a^	Deliveries^b^	SMM cases	Prevalence per 10 000 deliveries	OR (95% CI)^c^		
				Model 1	Model 2	Model 3
Nonredlined + exclusive	207 787	2499	120	1 [Reference]	1 [Reference]	1 [Reference]
Nonredlined + displacement	57 064	757	133	1.19 (1.08-1.32)	1.09 (0.98-1.21)	1.11 (1.00-1.23)
Nonredlined + gentrification	10 827	151	139	1.21 (1.00-1.47)	1.14 (0.94-1.39)	1.14 (0.94-1.38)
Redlined + exclusive	441 211	5919	134	1.17 (1.10-1.23)	1.13 (1.06-1.19)	1.12 (1.06-1.19)
Redlined + displacement	629 969	9376	149	1.32 (1.24-1.39)	1.18 (1.12-1.26)	1.21 (1.14-1.28)
Redlined + gentrification	207 979	3086	148	1.33 (1.24-1.42)	1.20 (1.12-1.29)	1.21 (1.13-1.29)

Abbreviations: OR, odds ratio; SMM, severe maternal morbidity.

^a^ Historically redlined neighborhoods received a Home Owners’ Loan Corporation (HOLC) grade of A or B, nonredlined neighborhoods a C or D. The Displacement and Gentrification Typology was used to define displacement (low income or susceptible to displacement, ongoing displacement of low-income households), gentrification (at risk of gentrification, early ongoing gentrification, advanced gentrification), and exclusive (stable moderate or mixed income, at risk of becoming exclusive, becoming exclusive, stable or advance exclusive).

^b^ Births outside of HOLC map coverage were not included in this study sample.

^c^ Model 1 was adjusted for age; model 2, age, education, and insurance type; model 3, age, education, insurance type, and parity.

Race and ethnicity did not modify the associations between redlining, gentrification, and SMM (*P* = .18). Sensitivity analysis assessing nontransfusion SMM showed similar results, with the effect sizes being slightly larger for statistically significant associations between residency in redlined areas undergoing displacement or gentrification (eTable 5 in Supplement 1). Furthermore, residency in a nonredlined neighborhood undergoing displacement was statistically significantly associated with SMM odds.

## Discussion

In a population-based cohort in California, we documented how past and present mechanisms that shape neighborhood material realities affect SMM. Living in a tract that experienced historical redlining and contemporary gentrification or displacement was associated with greater SMM odds when compared with living in an economically advantaged neighborhood unexposed to redlining. Descriptive findings also highlighted that racially and ethnically marginalized birthing people were disproportionately residing in census tracts that have experienced historical redlining and/or undergoing displacement and gentrification, which saw a higher SMM prevalence. Neighborhood history of past discriminatory policies, compounded by current dynamics of development and upheaval, may influence the risk of adverse pregnancy morbidity outcomes.

This study demonstrates the importance of understanding pregnancy outcomes within the context of historical processes that shape neighborhood conditions.^21,69^ Regardless of a neighborhood’s current gentrification status, living in redlined census tracts was associated with greater SMM odds compared with living in neighborhoods that experienced neither process. These findings align with prior studies documenting that the legacies of HOLC redlining remain connected with SMM, even after accounting for contemporary neighborhood deprivation, and also influence other related outcomes, including adverse birth outcomes.^17,18,20^ Our study builds on this evidence, emphasizing that living in a neighborhood with an upward trajectory from a redlined area to a socioeconomically advantaged neighborhood is insufficient to fully remedy historical disinvestment. In redlined and exclusive neighborhoods, historical housing discrimination may have resulted in the concentration of racially marginalized residents, and although some have been displaced, those who remain may encounter financial strain and barriers to accessing community-based resources.

Findings also build on evidence documenting the influence of gentrification on perinatal outcomes, by situating gentrification within the historical context of housing discrimination. Consistent with our hypothesis, living in a neighborhood that experienced the sequential processes of redlining and gentrification, as well as displacement, was associated with higher SMM odds, compared with living in a tract that did not experience these processes. Because the DGT assessment methodology emphasizes housing affordability, our findings align with prior work documenting that material deprivation and psychosocial stress from coping with rising housing costs may produce adverse SMM outcomes.^70,71^ Interventions to ensure housing affordability and curb displacement, such as rent control and rental assistance programs, may be vital in areas that have experienced housing discrimination.^22,69^ Prior studies found that neighborhood displacement and gentrification were associated with increased odds of SMM, and associations with other related outcomes were more mixed.^26,35,52,72,73^ When examining the influence of residing in nonredlined areas undergoing gentrification or displacement, the estimates were similar but the confidence intervals contained the null. A plethora of other policies, such as racially restrictive covenants and exclusionary zoning, may have contributed to the disinvestment in nonredlined neighborhoods that influenced their experience of displacement and gentrification, explaining the similar estimates. A limited number of census tracts in California were graded A and B while also undergoing displacement and gentrification, thus a very small study sample resided in nonredlining neighborhoods experiencing displacement and gentrification, further highlighting the connections between housing discrimination and contemporary neighborhood upheaval.

Although we did not find statistical evidence of effect measure modification by race and ethnicity, findings showed that American Indian and Alaskan Native and Black people in redlined tracts undergoing displacement or gentrification experienced the highest SMM prevalence, illuminating the injustices of populations contending with historical marginalization and present-day struggles, such as the housing crisis, while coping with high rates of adverse pregnancy outcomes.^74,75^ Notably, SMM prevalence was the lowest in nonredlined and exclusive tracts overall, as well as among American Indian and Alaskan Native, Hispanic, and White people, but not for Asian and Pacific Islander and Black individuals. In nonredlined and exclusive tracts, SMM were more prevalent among Black birthing people than White birthing people (2.1% vs 1.0%), an inequity suggesting that residing in a consistently well-resourced neighborhood may not confer the same degree of protection for all. Black individuals residing in these neighborhoods may contend with interpersonal discrimination, policing, and reduced access to resources, negatively impacting pregnancy outcomes.^76,77^

### Limitations

This study had several limitations. A major limitation is that HOLC redlining maps were only created for 8 cities due to city population size, resulting in the exclusion of the majority of California births from the analysis and reducing the generalizability of findings. Examining other historical housing discrimination measures in combination with gentrification would enable the inclusion of births in areas outside of HOLC coverage and strengthen evidence on the combined impact of past and present neighborhood disinvestment and upheaval.^12,13^ Our study design could not collect information on residency length or follow-up with displaced residents, which may have resulted in an underestimation of associations and masked heterogeneity in differential impact on those who moved into, stayed in, or were displaced from gentrifying neighborhoods. While we selected two 10-year assessment periods and implemented a lag to ensure temporality, the measurement of gentrification is sensitive to both the length and timing of the assessment period. A longitudinal study design can improve exposure classification and investigate differential impact based on residential history. Lastly, we defined neighborhoods using census tracts, but interventions designed to protect against displacement, such as the construction of affordable housing and rent control, may be implemented at the city or county level, and investigations at these scales may be informative.

## Conclusions

This study documented the impact of residency in a neighborhood with combined exposure to redlining and gentrification on SMM. To understand and address the enduring racial and ethnic inequities in the well-being of birthing people, it is imperative to contextualize the production of these inequities within historical drivers while also identifying contemporary sociopolitical mechanisms as key points of intervention.

## Notes

Supplement 1. **eTable 1.** Severe Maternal Morbidity Indicators, *International Classification of Diseases 9th and 10th Revision* Codes **eTable 2.** Urban Displacement Project’s Displacement and Gentrification Typology Exposure Assessment Methods **eTable 3.** Linkage of gentrification exposure measures with births in California, 2005-2018 **eTable 4.** Gentrification and Redlining Status of Census Tracts in California, 2005-2018 (N = 2344) **eTable 5.** Adjusted Odds Ratios of Non-Transfusion Severe Maternal Morbidity; California, 2005-2018 (n = 1 542 699) **eTable 6.** Adjusted Predicted Average Probabilities of Severe Maternal Morbidity Per 100 000 Births by Redlining-Gentrification; California, 2005-2018 **eFigure.** Analytic Sample Selection, California, 2005-2018

Supplement 2.
Data Sharing Statement
