# Supplementary material for: Historical Redlining, Contemporary Gentrification, and Severe Maternal Morbidity in California, 2005-2018
Source: JAMA Netw Open. 2024 Sep 23;7(9):e2429428. doi: 10.1001/jamanetworkopen.2024.29428 (PMC11420692; doi:10.1001/jamanetworkopen.2024.29428)
Supplement: Supplement 1. — eTable 1. Severe Maternal Morbidity Indicators, International Classification of Diseases 9th and 10th Revision Codes eTable 2. Urban Displacement Project’s Displacement and Gentrification Typology Exposure Assessment Methods eTable 3. Linkage of gentrification exposure measures with births in California, 2005-2018 eTable 4. Gentrification and Redlining Status of Census Tracts in California, 2005-2018 (N = 2344) eTable 5. Adjusted Odds Ratios of Non-Transfusion Severe Maternal Morbidity; California, 2005-2018 (n = 1 542 699) eTable 6. Adjusted Predicted Average Probabilities of Severe Maternal Morbidity Per 100 000 Births by Redlining-Gentrification; California, 2005-2018 eFigure. Analytic Sample Selection, California, 2005-2018 [file jamanetwopen-e2429428-s001.pdf]

Supplemental Online Content

Gao X, Morello-Frosch R, Nuru-Jeter AM, Snowden JM, Carmichael SL, Mujahid MS. Historical redlining, contemporary gentrification, and severe maternal morbidity in California, 2005-2018. *JAMA Netw Open.* 2024;7(9):e2429428. doi:10.1001/jamanetworkopen.2024.29428

eTable 1. Severe Maternal Morbidity Indicators, *International Classification of Diseases 9th and 10th Revision* Codes

eTable 2. Urban Displacement Project's Displacement and Gentrification Typology Exposure Assessment Methods

eTable 3. Linkage of gentrification exposure measures with births in California, 2005-2018

eTable 4. Gentrification and Redlining Status of Census Tracts in California, 2005-2018 (N = 2344)

eTable 5. Adjusted Odds Ratios of Non-Transfusion Severe Maternal Morbidity; California, 2005-2018 (n = 1 542 699)

eTable 6. Adjusted Predicted Average Probabilities of Severe Maternal Morbidity Per 100 000 Births by Redlining-Gentrification; California, 2005-2018

eFigure. Analytic Sample Selection, California, 2005-2018

This supplemental material has been provided by the authors to give readers additional information about their work.

© 2024 Gao X et al. *JAMA Network Open.*

eTable 1: Severe Maternal Morbidity Indicators, *International Classification of Diseases 9th and 10th Revision* Codes

Severe Maternal Morbidity Indicator	DX or PR	ICD-9	ICD-10
1. Acute myocardial infraction	DX	410.xx	I21.xx, I22.x
2. Aneurysm	DX	441.xx	I71.xx, I79.0
3. Acute renal failure	DX	584.5, 584.6, 584.7, 584.8, 584.9, 669.3x	N17.x, O90.4
4. Adult respiratory distress syndrome	DX	518.5x, 518.81 518.82, 518.84, 799.1	J80, J95.1, J95.2, J95.3, J95.82x, J96.0x, J96.2x, J96.9x, R06.03, R09.2
5. Amniotic fluid embolism	DX	673.1	O88.112, O88.113, O88.119, O88.12, O88.13
6. Cardiac arrest/ventricular fibrillation	DX	427.41, 427.42, 427.5	I46.x, I49.0x
7. Conversion of cardiac rhythm	PR	99.6x	5A2204Z, 5A12012
8. Disseminated intravascular coagulation	DX	286.6, 286.9, 641.3x, 666.3x	D65, D68.8, D68.9, O45.002, O45.003, O45.009, O45.012, O45.013, O45.019, O45.022, O45.023, O45.029, O45.092, O45.093, O45.099, O46.002, O46.003, O46.009, O46.012, O46.013, O46.019, O46.022, O46.023, O46.029, O46.092, O46.093, O46.099, O67.0, O72.3
9. Eclampsia	DX	642.6x	O15. X
10. Heart failure/arrest during surgery or procedure	DX	997.1	I97.12x, I97.13x, I97.710, I97.711
11. Puerperal cerebrovascular disorders	DX	046.3, 348.39, 362.34, 430.xx, 431.xx, 432.xx, 433.xx, 434.xx, 435.xx, 436xx, 437.xx, 671.5x, 674.0x, 997.02	A81.2, G45.x, G46.x, G93.49, H34.0x, I60.xx, I61.xx, I62.xx, I63.xx, I65.xx, I66.xx, I67.xx, I68.xx, O22.52, O22.53, I97.81x, I97.82x, O87.3
12. Pulmonary edema / Acute heart failure	DX	518.4, 428.1, 428.0, 428.21, 428.23, 428.3, 428.31, 428.33, 428.40, 428.41, 428.43, 428.9	J81.0, I50.1, I50.20, I50.21, I50.23, I50.30, I50.31, I50.33, I50.40, I50.41, I50.43, I50.810, I50.811, I50.813, I50.814, I50.82, I50.83, I50.84, I50.89, I50.9
13. Severe anesthesia complication	DX	668.0x, 668.1x, 668.2x, 995.4, 995.86	O29.112, O29.113, O29.119, O29.122, O29.123, O29.129, O29.192, O29.193, O29.199, O29.212, O29.213, O29.219, O29.292, O29.293, O29.299, O74.0, O74.1, O74.2, O74.3, O89.0x, O89.1, O89.2, T88.2XXA, T88.3XXA
14. Sepsis	DX	038.xx, 670.2x, 998.02, 995.91, 995.92, 785.52, 449	O85, R65.21, R65.20, T81.44XA, T81.12XA, I76, O86.04, A40.x, A41.x, A32.7
15. Shock	DX	669.1x, 785.50, 785.51, 785.59, 995.0, 998.0*, 998.00, 998.01, 998.09	O75.1, R57.x, T78.2XXA, T88.6 XXA, T81.10XA , T81.11XA, T81.19XA
16. Sickle cell disease with crisis	DX	282.42, 282.62, 282.64, 282.69, 289.52	D57.0x, D57.21x, D57.41x, D57.81x
17. Air and thrombotic embolism	DX	415.0, 415.1x, 673.0x, 673.2x, 673.3x, 673.8x	I26.01, I26.02, I26.09, I26.90, I26.92, I26.93, I26.94, I26.99, O88.012, O88.013, O88.019, O88.02, O88.03, O88.212, O88.213, O88.219, O88.22, O88.23, O88.312, O88.313, O88.319,

© 2024 Gao X et al. *JAMA Network Open.*

			O88.32, O88.33, O88.812, O88.813, O88.819, O88.82, O88.83, T80.0XXA
18. Blood products transfusion	PR	99.0x	30233H1, 30233L1, 30233K1, 30233M1, 30233N1, 30233P1, 30233R1, 30233T1, 30233H0, 30233L0, 30233K0, 30233M0, 30233N0, 30233P0, 30233R0, 30233T0, 30230H1, 30230L1, 30230K1, 30230M1, 30230N1, 30230P1, 30230R1, 30230T1, 30230H0, 30230L0, 30230K0, 30230M0, 30230N0, 30230P0, 30230R0, 30230T0, 30240H1, 30240L1, 30240K1, 30240M1, 30240N1, 30240P1, 30240R1, 30240T1, 30240H0, 30240L0, 30240K0, 30240M0, 30240N0, 30240P0, 30240R0, 30240T0, 30243H1, 30243L1, 30243K1, 30243M1, 30243N1, 30243P1, 30243R1, 30243T1, 30243H0, 30243L0, 30243K0, 30243M0, 30243N0, 30243P0, 30243R0, 30243T0, 30250H1, 30250L1, 30250K1, 30250M1, 30250N1, 30250P1, 30250R1, 30250T1, 30250H0, 30250L0, 30250K0, 30250M0, 30250N0, 30250P0, 30250R0, 30250T0, 30253H1, 30253L1, 30253K1, 30253M1, 30253N1, 30253P1, 30253R1, 30253T1, 30253H0, 30253L0, 30253K0, 30253M0, 30253N0, 30253P0, 30253R0, 30253T0, 30260H1, 30260L1, 30260K1, 30260M1, 30260N1, 30260P1, 30260R1, 30260T1, 30260H0, 30260L0, 30260K0, 30260M0, 30260N0, 30260P0, 30260R0, 30260T0, 30263H1, 30263L1, 30263K1, 30263M1, 30263N1, 30263P1, 30263R1, 30263T1, 30263H0, 30263L0, 30263K0, 30263M0, 30263N0, 30263P0, 30263R0, 30263T0
19. Hysterectomy	PR	68.39, 68.49, 68.59, 68.69, 68.79, 68.9x	0UT90ZZ, 0UT97ZL, 0UT97ZZ, 0UT90ZL
20. Temporary tracheostomy	PR	31.1	0B110F4, 0B113F4, 0B114F4
21. Ventilation	PR	96.70, 96.71, 96.72	5A1935Z, 5A1945Z, 5A1955Z

DX=diagnostic code
PR=procedure code

© 2024 Gao X et al. *JAMA Network Open.*

eTable 2: Urban Displacement Project's Displacement and Gentrification Typology Exposure Assessment Methods

Category		CRITERIA
Displacement	LOW INCOME/SUSCEPTIBLE TO DISPLACEMENT	• Low or mixed low-income tract by the end of the period
	ONGOING DISPLACEMENT OF LOW-INCOME HOUSEHOLDS	• Low or mixed low-income tract by the end of the period<br>• Absolute loss of low-income households during the period
Gentrification	AT RISK OF GENTRIFICATION	• Low income or mixed-low income tract by the end of the period<br>• Housing affordable to low or mixed-low income households by the end of the period<br>• Did not gentrify during 1990-2000 or during the period<br>• Marginal change in housing costs OR Zillow home or rental value increases in the 90th percentile during the period<br>• Local and nearby increases in rent were greater than the regional median during the period OR the end of period rent gap is greater than the regional median rent gap
	EARLY ONGOING GENTRIFICATION	• Low income or mixed-low income tract by the end of the period<br>• Housing affordable to moderate or mixed moderate-income households by the end of the period<br>• Increase or rapid increase in housing costs OR above regional median change in Zillow home or rental values during the period<br>• Gentrified between 1990-2000 or during the period
	ADVANCED GENTRIFICATION	• Moderate, mixed moderate, mixed high, high income tract by the end of the period<br>• Housing affordable to middle, high, mixed moderate, and mixed high-income households by the end of the period<br>• Marginal change or increase in housing cost<br>• Gentrified between 1990-2000 or during the period
Exclusive	STABLE MODERATE/MIXED INCOME	• Moderate, mixed-moderate, mixed-high, high income tract by the end of the period
	AT RISK OF BECOMING EXCLUSIVE	• Moderate, mixed-moderate, mixed-high, high income tract by the end of the period<br>• Housing affordable to middle, high, mixed-moderate, and mixed-high income households by the end of the period<br>• Marginal change or increase in housing cost
	BECOMING EXCLUSIVE	• Moderate, mixed-moderate, mixed-high, high income tract by the end of the period<br>• Housing affordable to middle, high, mixed-moderate, and mixed-high income households by the end of the period<br>• Rapid increase in housing costs<br>• Absolute loss of low-income households during the period<br>• Absolute loss of low-income households during the period

© 2024 Gao X et al. *JAMA Network Open.*

		• Median income higher at the end of the period compared to the beginning
	STABLE/ADVANCED EXCLUSIVE	• High-income tract in at the beginning of the period<br>• Affordable to high income household by the end of the period<br>• Marginal change, increase, or rapid increase in housing costs

© 2024 Gao X et al. *JAMA Network Open.*

eTable 3: Linkage of gentrification exposure measures with births in California, 2005-2018

	Exposure Data Year		Linked birth years
	Beginning of the period	End of the period	
Period 1	Decennial Census 2000	American Community Survey 5-Year Estimate: 2008-2012	Births between 2005 and 2011
Period 2	American Community Survey 5-Year Estimate: 2005-2009	American Community Survey 5-Year Estimate: 2015-2019	Births between 2012 and 2018

© 2024 Gao X et al. *JAMA Network Open.*

eTable 4: Gentrification and Redlining Status of Census Tracts in California, 2005-2018 (N=2,344)

		HOLC Grade			
		A (N=120)	B (N=445)	C (N=1123)	D (N=656)
Typology	Exclusive (N=1,176)	4.7%	14.0%	21.5%	10.0%
	Displacement (N=884)	0.5%	4.2%	19.5%	13.6%
	Gentrification (N=284)	0.0%	0.8%	6.9%	4.4%

cell percentage is displayed

© 2024 Gao X et al. *JAMA Network Open.*

eTable 5. Adjusted Odds Ratios of Non-Transfusion Severe Maternal Morbidity; California, 2005-2018 (n = 1,542,699)

	Deliveries	SMM Cases	Prevalence Per 10,000 Deliveries	Model 1 OR (95% CI)	Model 2 OR (95% CI)	Model 3 OR (95% CI)
**Redlining + Gentrification**						
Non-redlined + Exclusive	206,375	1,087	53	--	--	--
Non-redlined + Displacement	56,651	344	61	1.30 (1.14-1.49)	1.17 (1.02-1.34)	1.19 (1.03-1.36)
Non-redlined + Gentrification	10,737	61	57	1.16 (0.88-1.54)	1.09 (0.82-1.43)	1.07 (0.81-1.42)
Redlined + Exclusive	437,862	2,570	59	1.18 (1.09-1.28)	1.14 (1.05-1.23)	1.13 (1.05-1.22)
Redlined + Displacement	624,776	4,183	67	1.45 (1.35-1.57)	1.28 (1.18-1.38)	1.30 (1.20-1.41)
Redlined + Gentrification	206,298	1,405	68	1.46 (1.34-1.60)	1.29 (1.18-1.42)	1.30 (1.19-1.43)

Model 1: adjusted for age
Model 2: adjusted for covariates in Model 1, education, insurance type
Model 3: adjusted for covariates in Model 2 and parity
Historical redlining: Redlined = Home Owners' Loan Corporation (HOLC) Grade A or B, Non-redlined= HOLC Grade C or D
Displacement and Gentrification Typology: Displacement (low income/susceptible to displacement, ongoing displacement of low-income households), gentrification (at risk of gentrification, early ongoing gentrification, advanced gentrification), and exclusive (stable moderate/mixed income, at risk of becoming exclusive, becoming exclusive, stable/advance exclusive)
SMM: severe maternal morbidity
Births outside of HOLC map coverage were not included in this study sample

© 2024 Gao X et al. *JAMA Network Open.*

eTable 6: Adjusted Predicted Average Probabilities of Severe Maternal Morbidity Per 100,000 Births by Redlining-Gentrification; California, 2005-2018

	Overall (Per 10,000 births) (95% CI)	Asian/Pacific Islander (Per 10,000 births) (95% CI)	Black (Per 10,000 births) (95% CI)	Hispanic (Per 10,000 births) (95% CI)	White (Per 10,000 births) (95% CI)
**Redlining + Gentrification**					
Non-redlined + Exclusive	121 (116-127)	139 (128-151)	198 (171-226)	119 (109-130)	101 (94-108)
Non-redlined + Displacement	134 (122-146)	112 (74-150)	192 (157-227)	129 (116-143)	121 (94-151)
Non-redlined + Gentrification	138 (112-164)	134 (87-180)	192 (85-299)	138 (103-172)	128 (76-180)
Redlined + Exclusive	136 (132-140)	138 (129-146)	197 (178-215)	139 (132-145)	116 (110-123)
Redlined + Displacement	146 (142-150)	140 (129-150)	217 (205-229)	142 (137-146)	118 (108-129)
Redlined + Gentrification	147 (140-153)	138 (124-153)	212 (188-236)	144 (136-151)	127 (110-143)

Model adjusted for age, education, insurance, and parity
Historical redlining: Redlined = Home Owners' Loan Corporation (HOLC) Grade A or B, Non-redlined= HOLC Grade C or D
Displacement and Gentrification Typology: Displacement (low income/susceptible to displacement, ongoing displacement of low-income households), gentrification (at risk of gentrification, early ongoing gentrification, advanced gentrification), and exclusive (stable moderate/mixed income, at risk of becoming exclusive, becoming exclusive, stable/advance exclusive)
Births outside of HOLC map coverage were not included in this study sample
American Indian/Alaskan Native & Other racial groups not included due to insufficient sample size to calculate predicted probabilities

© 2024 Gao X et al. *JAMA Network Open.*

eFigure 1: Analytic Sample Selection, California, 2005-2018

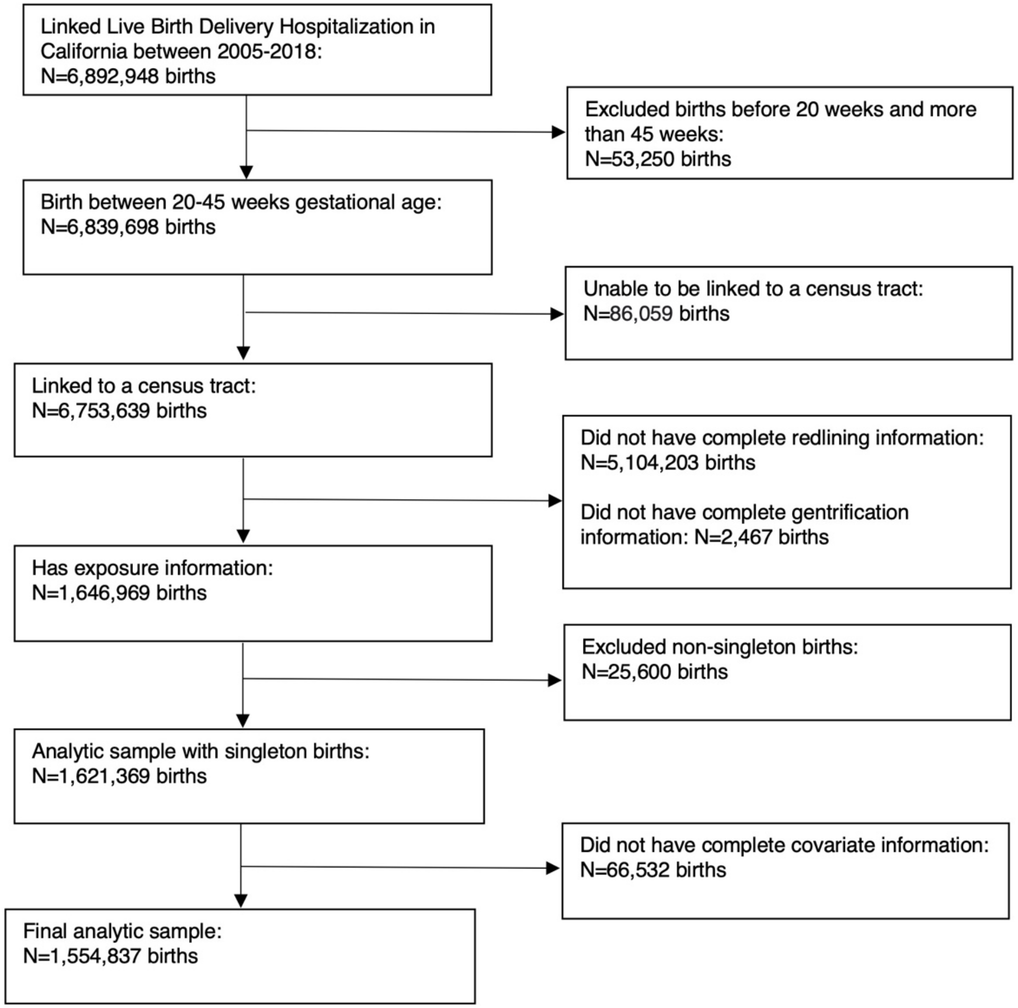

© 2024 Gao X et al. *JAMA Network Open.*
