# Supplementary material for: Historical Redlining, Contemporary Gentrification, and Severe Maternal Morbidity in California, 2005-2018
Source: JAMA Netw Open. 2024 Sep 23;7(9):e2429428. doi: 10.1001/jamanetworkopen.2024.29428 (PMC11420692; doi:10.1001/jamanetworkopen.2024.29428)
Supplement: Supplement 2. — Data Sharing Statement [file jamanetwopen-e2429428-s002.pdf]

Data Sharing Statement

Gao. Redlining, Gentrification, and Severe Maternal Morbidity in California, 2005-2018. *JAMA Netw Open*. Published September 23, 2024. doi:10.1001/jamanetworkopen.2024.29428

Data
Data available: No
